# Mitochondrial haplogroup H1 is protective for ischemic stroke in Portuguese patients

**DOI:** 10.1186/1471-2350-9-57

**Published:** 2008-07-01

**Authors:** Alexandra Rosa, Benedita V Fonseca, Tiago Krug, Helena Manso, Liliana Gouveia, Isabel Albergaria, Gisela Gaspar, Manuel Correia, Miguel Viana-Baptista, Rita Moiron Simões, Amélia Nogueira Pinto, Ricardo Taipa, Carla Ferreira, João Ramalho Fontes, Mário Rui Silva, João Paulo Gabriel, Ilda Matos, Gabriela Lopes, José M Ferro, Astrid M Vicente, Sofia A Oliveira

**Affiliations:** 1 Instituto Gulbenkian de Ciência, Oeiras, Portugal; 2 Instituto Nacional de Saúde Dr. Ricardo Jorge, Lisboa, Portugal; 3 Serviço de Neurologia, Hospital de Santa Maria, Lisboa, Portugal; 4 Serviço de Neurologia, Hospital Geral de Santo António, Porto, Portugal; 5 Serviço de Neurologia, Hospital Garcia de Orta, Almada, Portugal; 6 Serviço de Neurologia, Hospital Fernando Fonseca, Amadora, Portugal; 7 Serviço de Neurologia, Hospital São Marcos, Braga, Portugal; 8 Serviço de Neurologia, Hospital de São Pedro, Vila Real, Portugal; 9 Serviço de Neurologia, Hospital Distrital de Mirandela, Mirandela, Portugal

## Abstract

**Background:**

The genetic contribution to stroke is well established but it has proven difficult to identify the genes and the disease-associated alleles mediating this effect, possibly because only nuclear genes have been intensely investigated so far. Mitochondrial DNA (mtDNA) has been implicated in several disorders having stroke as one of its clinical manifestations. The aim of this case-control study was to assess the contribution of mtDNA polymorphisms and haplogroups to ischemic stroke risk.

**Methods:**

We genotyped 19 mtDNA single nucleotide polymorphisms (SNPs) defining the major European haplogroups in 534 ischemic stroke patients and 499 controls collected in Portugal, and tested their allelic and haplogroup association with ischemic stroke risk.

**Results:**

Haplogroup H1 was found to be significantly less frequent in stroke patients than in controls (OR = 0.61, 95% CI = 0.45–0.83, p = 0.001), when comparing each clade against all other haplogroups pooled together. Conversely, the pre-HV/HV and U mtDNA lineages emerge as potential genetic factors conferring risk for stroke (OR = 3.14, 95% CI = 1.41–7.01, p = 0.003, and OR = 2.87, 95% CI = 1.13–7.28, p = 0.021, respectively). SNPs m.3010G>A, m.7028C>T and m.11719G>A strongly influence ischemic stroke risk, their allelic state in haplogroup H1 corroborating its protective effect.

**Conclusion:**

Our data suggests that mitochondrial haplogroup H1 has an impact on ischemic stroke risk in a Portuguese sample.

## Background

**Figure 1:**
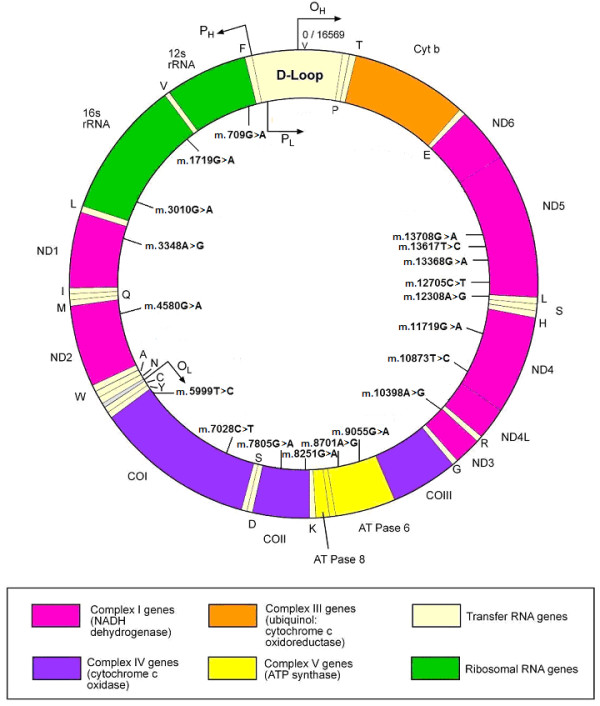
**Genomic localization of the investigated markers within the human mitochondrial DNA molecule**. The genetic location of mtDNA markers genotyped in the present study is indicated in the inner circle. H and L stand for heavy and light strands, respectively, given the asymmetric distribution of G and C nucleotides, with H being the G-rich strand. The seven complex I subunits (ND1, 2, 3, 4L, 4, 5 and 6), one complex III subunit (Cyt b), three complex IV subunits (COI, COII, and COIII), two complex V subunits (ATPases 6 and 8), two ribosomal RNAs (12S and 16S rRNAs), 22 tRNAs and D-loop regions are shown. Gene products encoded by the L-strand are shown in the inner circle (one letter code) while the products of the H-strand are shown in the outer circle. Arrows indicate the locations of promoters P_L _and P_H _for the transcription and replication origin O_H_. Adapted from MITOMAP [2].

Stroke is a complex disorder resulting from the interplay of genetics and environment, and genes potentially having an impact on disease pathogenesis (e.g. genes involved in hemostasis), intermediate phenotypes (e.g. atherosclerosis) or clinical risk factors (e.g. blood pressure regulation) have been tested for association with stroke risk [1]. Mostly nuclear genes have been intensively investigated thus far, while the role of the mitochondrial genome has been neglected. Mitochondria are extranuclear organelles whose primary function is the production of ATP through the oxidative phosphorylation (OXPHOS) respiratory chain. They also play a decisive role in intracellular signaling, metabolic pathways such as Krebs' or tricarboxylic acid cycle and the metabolism of amino acids, lipids, cholesterol and steroids. Mitochondrial function is required for normal vascular cell growth and function, and its dysfunction can result in apoptosis, favoring atherosclerotic plaque rupture. mtDNA is maternally inherited, does not recombine and exhibits high mutation and fixation rates, therefore making it an important tool in phylogenetics. Human mtDNA is a haploid, circular molecule of approximately 16,600 nucleotides encoding for thirteen OXPHOS polypeptides, twenty-two transfer RNAs and two ribosomal RNAs (Figure 1) [2]. Particular combinations of certain polymorphisms define mitochondrial haplogroups and subclades (Table 1), which tend to be associated to broad geographic areas and/or populations [3].

**Table 1:** Type of investigated mitochondrial markers and haplogroup determination.

	Mitochondrial Polymorphism (SNP Type)*																		
	
**Haplogroup**	m.709G>A (ncod)	m.1719G>A (ncod)	m.3010G>A (ncod)	m.3348A>G (syn)	m.4580G>A (syn)	m.5999T>C (syn)	m.7028C>T (syn)	m.7805G>A (p.V74I)	m.8251G>A (syn)	m.8701A>G (p.T59A)	m.9055G>A (p.A177T)	m.10398A>G (p.T114A)	m.10873T>C (syn)	m.11719G>A (syn)	m.12308A>G (ncod)	m.12705C>T (syn)	m.13368G>A (syn)	m.13617T>C (syn)	m.13708G>A (p.A458T)
H							**C**							**G**					
H1			**A**				**C**							**G**					
V					**A**		T							**G**					
pre-HV/HV					**G**		**T**							**G**					
J		G										**G**		**A**	**A**	**C**			**A**
J1b		G	**A**									**G**		**A**	**A**	**C**			**A**
T	**A**											**A**		A		C	**A**		
U														A	**G**	C			
U4						**C**								A	**G**	C			
U5														A	**G**	C		**C**	
U6a				**G**				**A**						A	**G**	C			
K1											**A**	**G**			**G**				
I	**G**	**A**							**A**	A		**G**	T			T			
X2b	**G**	**A**							**G**	A		**A**	T			T			**A**
W	**A**	**G**							**A**	A		**A**	T			T			
L										**G**		**G**	**C**			T			

Each haplogroup was determined by the combination of bolded alleles, and the alleles not bolded aided in the phylogenetic assignment. The polymorphisms are named after their base pair position and alleles.

*ncod: non-coding SNP; syn: synonymous SNP; amino acid substitutions are indicated for non-synonymous SNPs.

Particular variants of the mitochondrial genome have been linked to aging [4,5], the strongest risk factor for stroke, and to several neurological and vascular disorders. Among the best-known examples of a mitochondrial disorder is that of MELAS (MIM: 540000), a mitochondrial encephalopathy characterized by lactic acidosis and stroke-like episodes. This syndrome is caused by the m.3243A>G mutation, an A to G transition at mtDNA nucleotide position 3243 [6,7]. Leber's hereditary optic neuropathy (LHON, MIM: 535000), a vascular disease of the optic disc, is also caused by mtDNA mutations that lead to respiratory chain dysfunction [8]. Interestingly, the phylogenetic background of haplogroup J influences the clinical penetrance and expression of the m.11778G>A and m.14484T>C primary LHON mutations [9,10]. This exemplifies how, although defined on the basis of evolutionarily neutral polymorphisms, common mtDNA variation of phylogenetic relevance assumes a functional role on the expression of particular complex traits. mtDNA variation has been associated with non-Mendelian and non-maternally inherited complex disorders such as Parkinson's disease [11], Alzheimer's disease [12], myocardial infarction [13], obesity [14], occipital stroke in migraine [15,16], and mean intima-media thickness of bilateral carotid arteries [17]. Increased mitochondrial oxidative stress and dysfunction has been linked to many ischemic stroke risk factors, including hypertension [18], diabetes [19], inflammation [20], plaque rupture [20], tobacco smoke and alcohol exposure [21]. The goal of the present study was to determine whether mtDNA SNPs or haplogroups predispose to ischemic stroke in a large cohort of Portuguese patients and controls.

## Methods

### Study subjects

Five hundred thirty four unrelated patients with a clinical diagnosis of ischemic stroke, who were under the age of 65 at stroke onset, were recruited through Neurology and Internal Medicine Departments throughout Portugal. Stroke was defined by the presence of a new focal neurological deficit, with an acute onset and symptoms and signs persisting for more than 24 hours, and was confirmed by Computed Tomography Scan (97% of cases) and/or Magnetic Resonance Imaging (in 25% of patients) [22]. All patients were seen and all neuroradiology tests were reviewed by study neurologists. Trauma, tumors, infection and other causes of neurological deficit were excluded.

Data collection forms were developed for this study that included extensive clinical information such as stroke characteristics, general clinical observation, neurological symptoms and signs, complications and interventions during hospitalization and situation at discharge. Data was also collected on relevant lifestyle aspects and previous clinical risk factors.

Four hundred ninety nine unrelated healthy individuals were included in this study as a control sample population. Since stroke is a late-onset disease, the control group was selected from a group of healthy volunteers with a higher mean age than the case group, thus minimizing the chances for mis-classification as "stroke-free". Control individuals were verified to be free of stroke by direct interview before recruitment, but no brain imaging studies were performed. The interview also included questions on established clinical and life-style risk factors for stroke.

The principal demographic and clinical characteristics and frequency of risk factors of this study sample are shown in Table 2. The research protocol was approved by the ethics committees of participating institutions, and participants were informed of the study and provided informed consent.

**Table 2:** General characteristics of the ischemic stroke case-control study sample

**Characteristic**	**Controls**	**Cases**	P-value*
N	499	534	
Sex (n/N, %male)	230/499 (46.1)	336/534 (62.9)	< 10^-4^
Age-at-examination (mean ± SD, years)	62.9 ± 6.9	52.1 ± 9.4	< 10^-4^
Age-at-onset (mean ± SD, years)	-	51.4 ± 9.5	-
Risk factors (n/N, %)			
Hypertension (> 140–85 mmHg)	183/482 (38.0)	270/478 (56.5)	< 10^-4^
Hypercholestrolemia (> 200 mg/dL)	309/489 (63.2)	310/496 (62.5)	0.654
Hypertriglycemia (> 200 mg/dL)	64/411 (15.6)	38/215 (17.7)	0.508
Diabetes	54/470 (11.5)	88/509 (17.3)	0.007
Ever smoking	127/481 (26.4)	259/526 (49.2)	< 10^-4^
Ever drinking	202/474 (42.6)	305/527 (57.9)	< 10^-4^

*P-value of an unpaired Student's t test or a chi-square test for quantitative and qualitative data, respectively. SD: standard deviation.

### SNP selection and haplogroup definition

We studied nineteen mtDNA SNPs (Figure 1 and Table 1) of phylogenetic relevance for classifying the Portuguese mitochondrial haplogroup variation which includes the most prevalent West Eurasian haplogroups (H, H1, V, pre-HV/HV, J, J1b, T, U, U4, U5, K1, I, X2b, and W), as well as some African haplogroups (U6a and L) more frequent in Portugal and in the Iberian Peninsula than in other European countries [23,24]. Haplogroups and their subclades, which show different frequencies and distributions in human populations, are defined by the combination of multiple markers (Table 1), embracing the information from the whole set of branches of the mtDNA tree rather than the status at any single point mutation. The nomenclature of clades follows Torroni et al. [25], Richards et al. [26], and Macaulay et al. [27]. The "Other" haplogroup category in Table 3 (19 controls and 20 patients) includes individuals whose haplogroups could not be assigned to the clades in Table 1.

**Table 3:** Results of mitochondrial haplogroup association testing with ischemic stroke risk.

	**Number of Individuals (%)**		**Chi-square Test**		**Logistic Regression Model**	
			
**Haplogroup**	**Controls**	**Patients**	**P-value**	**OR [95% CI]**	**P-value**	**OR [95% CI]**
H	118 (23.6)	116 (21.7)	0.454		0.432	
H1	125 (25.1)	91 (17.0)	**0.001**	0.61 [0.45–0.83]	**0.007**	0.57 [0.38–0.85]
V	13 (2.6)	12 (2.2)	0.707		0.861	
pre-HV/HV	8 (1.6)	26 (4.9)	**0.003**	3.14 [1.41–7.01]	**0.008**	4.68 [1.51–14.54]
J	9 (1.8)	9 (1.7)	0.883		0.122	
J1b	23 (4.6)	24 (4.5)	0.928		0.862	
T	56 (11.2)	65 (12.2)	0.637		0.249	
U	6 (1.2)	18 (3.4)	**0.021**	2.87 [1.13–7.28]	**0.038**	4.01 [1.08–14.90]
U4	13 (2.6)	16 (3.0)	0.705		0.286	
U5	24 (4.0)	34 (6.3)	0.278		**0.048**	2.17 [1.01–4.67]
U6a	10 (2.0)	10 (1.9)	0.877		0.71	
K1	27 (5.4)	28 (5.2)	0.903		0.184	
I	10 (2.0)	11 (2.1)	0.950		0.500	
X2b	7 (1.4)	9 (1.7)	0.714		0.789	
W	6 (1.2)	11 (2.1)	0.279		0.394	
L	25 (5.0)	34 (6.4)	0.348		0.913	
Other*	19 (3.8)	20 (3.7)	-		-	

Significant uncorrected P-values (< 0.05) are highlighted in bold. Crude and adjusted odds ratios (OR) and 95% confidence intervals (CI) are shown only for significantly associated haplogroups.

*Given that the "Other" class includes an assortment of lineages not phylogeneticaly correlated, association tests were not performed.

### Genotyping

Genomic DNA was extracted from whole blood samples using the NucleoSpin Blood XL kit (Macherey-Nagel; Düren, Germany) or a salting out procedure. SNPs were genotyped using Sequenom's iPlex assay (primer extension of multiplex products with detection by matrix-assisted laser desorption/ionization time-of-flight mass spectrometry) following manufacturer's protocol and detected in a Sequenom MassArray K2 platform. The primer sequences are available upon request and were designed using Sequenom's (San Diego, USA) MassARRAY^® ^Assay Design 3.0 software according to the Cambridge reference sequence [2]. Extensive quality control was performed using eight HapMap controls of diverse ethnic affiliation, sample duplication within and across plates, non-Mendelian maternal inheritance check in three large pedigrees, and a minimum of 90% call rate. Genotype determinations were performed blinded to affection status. 0.2% of all calls were heterozygous, most likely due to mtDNA heteroplasmy, and these were not included in the analyses.

### Statistical analysis

An unpaired Student's *t *test and a χ^2 ^test were used to compare quantitative and qualitative clinical and demographic data, respectively, between cases and controls. χ^2 ^tests were performed to explore the association of each mtDNA SNP and haplogroup with stroke risk. For haplogroup analyses, we compared each haplogroup with all other haplogroups pooled together. To adjust the association analysis for confounding factors, age-at-examination, hypertension, diabetes and ever smoking were included as covariates in multivariate logistic regression with backward elimination of risk factors. The interaction *i *among covariates in regression models was not strong (-0.5 <*i *< 0.5). Logistic regressions were performed using the R freeware [28]. Odds ratios (ORs) and their associated 95% confidence intervals (CIs) were uncorrected for confounding variables in the χ^2 ^tests and corrected for covariates in regression models. Results were considered significant below the conventional level of 0.05. Since some of the markers are in linkage disequilibrium and the haplogroup comparisons are not independent, we did not perform corrections for multiple testing and uncorrected p-values are reported.

## Results

Table 2 shows the general characteristics of our dataset. Since stroke is a very common late-onset disorder, we chose to have the control group significantly older than the case group to minimize misclassification biases of control individuals. Male to female ratio, hypertension, diabetes, ever smoking, and ever drinking were significantly higher in ischemic stroke patients than in controls, and the effects of these potentially confounding variables were accounted for in the multivariate logistic regressions with backward elimination of risk factors. Our group of patients has a similar risk factor profile than previously described older groups of ischemic stroke cases with similar male to female ratios [29,30], and therefore can be considered representative of the general ischemic stroke population.

The mtDNA haplogroup distribution in the control group (Table 3) was in agreement with previously published data on a similar Portuguese normal population [23,24], with 8.3–9.9% of the individuals having mtDNA haplogroups characteristic of African populations (L and U6). With the genotyped SNPs, a haplogroup could not be assigned to an almost equal percentage of individuals in the control and patient groups (3.8 and 3.7%, respectively, classified as "Others" in Table 3), which again is in concordance with other studies using equivalent approaches [11,31]. These individuals have either an ambiguous SNP-profile or belong to rare Eurasian haplogroups (e.g. R, Z, M). The fact that L, U6, and "Others" haplogroup categories are present in equivalent proportions in cases (12.0%) and controls (10.8%) (Table 3) further suggests that our dataset was well matched for ethnicity and lacks significant substructure.

**Figure 2:**
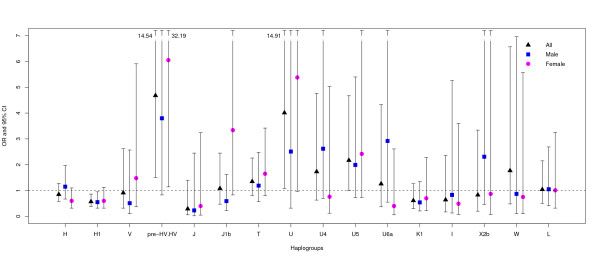
**Logistic regression odds ratios and confidence intervals (CIs) for mtDNA haplogroup association with ischemic stroke risk**. Bars indicate 95% CIs and are shown as dotted lines when the upper confidence limit (CL) is over 7. The upper CL is indicated when it is over 7 and the respective lower CL is greater than 1.

**Figure 3:**
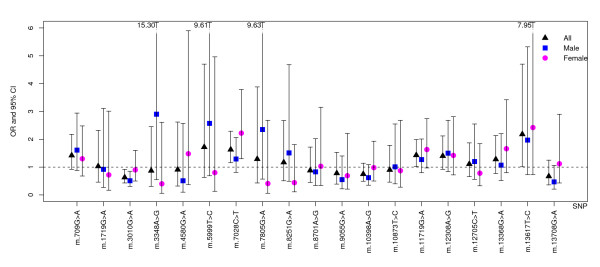
**Logistic regression odds ratios and confidence intervals (CIs) for mtDNA SNPs association with ischemic stroke risk**. Bars indicate 95% CIs and are shown as dotted lines when the upper confidence limit (CL) is over 6. The upper CL is indicated when it is greater than 6.

Results of crude and adjusted association analyses of mitochondrial haplogroups are shown in Figure 2 and Table 3, and those of single-markers are presented in Figure 3 and Table 4. Sub-haplogroup H1 was found to be significantly less frequent in ischemic stroke patients than in controls when comparing each clade against all others pooled together (χ^2 ^test OR = 0.61, 95%CI = 0.45–0.83, p = 0.001); when significant risk factors were included in the model as covariates (age-at-examination, hypertension, diabetes and ever smoking), the association remained significant (logistic regression OR = 0.57, 95%CI = 0.38–0.85, p = 0.007). The sub-haplogroup H1 is thus protective for ischemic stroke in this dataset. SNPs m.3010G>A, m.7028C>T and m.11719G>A, which together define H1, were found to consistently influence the risk for ischemic stroke in both the uncorrected χ^2 ^test and the logistic regression model, their allelic state in H1 corroborating its protective effect. Stratification by sex revealed that the crude association of haplogroup H1 is quite consistent among males (OR = 0.65, 95%CI = 0.43–0.98, p = 0.038) and females (OR = 0.58, 95%CI = 0.37–0.93, p = 0.021), even though it did not reach significance in females when adjusted for co-variates (Figure 2), most likely due to the small sample size.

**Table 4:** Results of mitochondrial SNP association testing with ischemic stroke risk. Significant uncorrected P-values (< 0.05) are highlighted in bold. Crude and adjusted odds ratios (OR) and 95% confidence intervals (CI) are shown only for significantly associated polymorphisms.

	**Number of Individuals (%)**		**Chi-square Test**		**Logistic Regression Model**	
			
SNPs*	**Controls**	**Patients**	**P-value**	**OR [95% CI]**	**P-value**	**OR [95% CI]**
m.709G>A	91 (18.3)	101 (19.1)	0.760		0.113	
m.1719G>A	21 (4.3)	31 (5.9)	0.239		0.942	
m.3010G>A	156 (31.3)	123 (23.0)	**0.003**	0.66 [0.50–0.87]	**0.016**	0.63 [0.43–0.92]
m.3348A>G	15 (3.0)	11 (2.1)	0.329		0.794	
m.4580G>A	13 (2.6)	12 (2.2)	0.704		0.860	
m.5999T>C	13 (2.6)	17 (3.2)	0.596		0.293	
m.7028C>T	248 (50.5)	307 (59.7)	**0.003**	1.45 [1.13–1.87]	**0.005**	1.63 [1.16–2.29]
m.7805G>A	11 (2.2)	12 (2.3)	0.960		0.656	
m.8251G>A	23 (4.6)	28 (5.3)	0.620		0.706	
m.8701A>G	33 (6.7)	41 (7.7)	0.530		0.714	
m.9055G>A	30 (6.0)	34 (6.4)	0.820		0.464	
m.10398A>G	97 (19.5)	111 (20.9)	0.591		0.178	
m.10873T>C	30 (6.4)	39 (7.5)	0.519		0.771	
m.11719G>A	235 (47.1)	289 (54.1)	**0.024**	1.33 [1.04–1.69]	**0.037**	1.43 [1.02–1.99]
m.12308A>G	92 (18.5)	114 (21.4)	0.255		0.118	
m.12705C>T	53 (11.6)	73 (14.5)	0.186		0.683	
m.13368G>A	57 (11.6)	66 (12.5)	0.679		0.337	
m.13617T>C	24 (4.8)	34 (6.3)	0.278		**0.047**	2.18 [1.01–4.70]
m.13708G>A	45 (9.0)	47 (8.9)	0.925		0.209	

*As an example, m.709G>A stands for a G to A transition at mtDNA nucleotide position 709. The number of individuals and test statistics refer to the derived (second) allele (A in this example).

Conversely, the pre-HV/HV, also known as R0 [32] (χ^2 ^test OR = 3.14, 95%CI = 1.41–7.01, p = 0.003; logistic regression OR = 4.68; 95%CI = 1.51–14.54, p = 0.008), and U (χ^2 ^test OR = 2.87, 95%CI = 1.13–7.28, p = 0.021; logistic regression OR = 4.01, 95%CI = 1.08–14.90, p = 0.038) mtDNA lineages emerge as potential genetic factors conferring risk for stroke (Figure 2 and Table 3). The relatively rare U5 sub-clade and its defining polymorphism m.13617T>C showed a trend for association with stroke risk only with the logistic regression test (OR = 2.17, 95%CI = 1.01–4.67, p = 0.048, and OR = 2.18; 95%CI = 1.01–4.70, p = 0.047, respectively).

## Discussion

To the best of our knowledge, this is the first comprehensive association study of mtDNA variation with ischemic stroke risk in an European population. In a large population sample of ethnically-matched cases and controls, we found that haplogroup H1 is protective while haplogroups pre-HV/HV and U increase risk for ischemic stroke. Since these haplogroups are defined by the combination of several polymorphisms also present in other clades (e.g. allele A of m.3010G>A is a phylogenetic marker of subclades H1 and J1b), the observed haplogroup associations cannot be attributed to particular SNPs, but instead to their precise arrangement. To exclude the possibility that the observed associations are due to population stratification with study participants of African or non-West Eurasian ancestries, we performed the statistical analyses in the overall dataset excluding the individuals with haplogroups U6a, L, and "Others" (54 controls and 64 patients), and obtained the same associations (data not shown). Unlike H1, the pre-HV/HV, U and U5 haplogroups were found in a small number of individuals, and therefore their association with stroke risk is only suggestive. Low counts tend to inflate the qui-square values and lead to false-positive results. We did not study the association of mtDNA with stroke subtypes since a much larger sample size would be required to have a representative number of individuals in each subtype and haplogroup category. Stratification by sex was performed as there are clear differences between male and female ischemic patients [33], and some of the associations (e.g. adjusted association of H1 in females) most likely did not reach statistical significance due to the relatively small sample sizes.

Earlier studies have addressed the contribution of mtDNA variation to stroke susceptibility. The m.12308A>G polymorphism defining haplogroups U and K, previously associated with occipital stroke in migraine [15,16] and suggested to increase the risk of developing stroke in MELAS patients with the m.3243A>G mutation [34], was not associated with ischemic stroke in our dataset. However, an association of the U5 subcluster with migrainous stroke has been reported [16] and is consistent with our tentative association of the U5 haplogroup and its defining m.13617T>C polymorphism with ischemic stroke. m.5178C>A, associated with aging [4] and cerebrovascular disorders (cerebral hemorrhage or infarction) in a small Japanese case-control sample [35] and with intima-media thickness in carotid arteries of Japanese type 2 diabetic individuals [17], could not be investigated here as it is Asian-specific [36]. Haplogroup A, unlike its defining polymorphisms m.663A>G in the 12S rRNA gene and m.8794C>T in the ATPase 6 gene, was recently found associated with atherothrombotic cerebral infarction in 440 Japanese females after adjustement for significant co-variates [37]. None of the three SNPs we studied in the 12S rRNA and ATPase 6 genes (m.709G>A, m.8701A>G, and m.9055G>A) were associated with ischemic stroke, suggesting that haplogroup A, but not its defining SNPs individually or other SNPs in the same genes, may constitute a risk factor for stroke in Japanese. Finally, we did not try to replicate the reported association with lacunar cerebral infarction of the m.16189T>C variant in the mtDNA hypervariable region [38] as we only investigated SNPs in the coding region and this polymorphism is not restricted to any particular haplogroup [39,40]. These discrepancies among reports highlight: i) the difficulty of finding reproducible mitochondrial genome associations with disease due to the continent-specificity of some mtDNA SNPs and clades, and ii) the necessity of performing association studies in very large samples so that even uncommon haplogroups are represented by a sufficient number of individuals. A power analysis of mitochondrial haplogroup association studies such as the present one (investigating 17 haplotypes) reveals that a sample of size similar to ours (515 cases and 515 controls) only provides 50% power to detect a change in haplogroup frequency from 0.251 in controls to 0.17 in cases (as observed here for H1) at a significance level of 0.05 [41]. Even though we only had 50% power, we detected an association of H1 at a significance level of 0.001, and this association would survive a Bonferroni correction for the seventeen crude or adjusted association tests performed for haplogroups, suggesting that it is an important association. Much larger cohorts are required for less common clades or finer changes in haplogroup frequency, and therefore the present study provides preliminary evidence of association that requires further validation in independent cohorts.

Although the polymorphisms that characterize the phylogeny are thought to be evolutionarily neutral, they may cause subtle alterations in the encoded transcripts or proteins, which collectively and over time, influence the risk of a stroke event. Given that stroke is mostly a late-onset disorder, it does not affect the successful transmission of mtDNA alleles and their fixation in the population. Additionally, several reports have documented the tissue-specific accumulation of mitochondrial deletions with aging [42,43], and it is conceivable that mtDNA polymorphisms or haplogroups which are neutral under normal circumstances become advantageous in post-mitotic tissues in the presence of acquired mutations.

The associated m.3010G>A non-coding polymorphism, located in the conserved 3' end of the 16S rRNA gene, lies near non-coding point mutations known to confer resistance to chloramphenicol, a prokaryotic and mitochondrial protein synthesis inhibitor [44]. The synonymous m.7028C>T transition is located in the cytochrome c oxidase (COX) subunit I gene (COI) of complex IV. This protein complex is the terminal enzyme of the respiratory chain, which collects electrons from reduced cytochrome c and catalyzes the reduction of oxygen to water, and consists of 13 polypeptide subunits, 3 of which are mtDNA-encoded. m.11719G>A is a synonymous SNP in the ND4 gene. ND4 gene product is a subunit of the respiratory complex I which accepts electrons from NADH, transfers them to ubiquinone and uses the energy released to pump protons across the mitochondrial inner membrane. A mutation in ND4 (m.11778G>A) causing an arginine to histidine change at amino acid 340 [MIM 516003.0001] accounts for over 50% and 90% of all LHON cases among Caucasians and Asians, respectively. Interestingly, the penetrance of this mutation is higher within a J haplogroup background, but its effect is most prominent on the J2 subclade [8,9]. The physical proximity of the associated polymorphism in ND4 to known mutations suggests that it lies in or close to important functional domains and has the potential to alter the protein's function.

It is interesting to notice that the majority of polymorphisms associated with stroke risk in the present report are localized in complexes I and IV, whose deficiencies are the most frequently observed abnormalities of the OXPHOS system. It would be of great interest to assess if stroke patients display complex I and IV deficits relative to matched controls, prior to their first stroke, and to identify phenotypic differences among haplogroups using transmitochondrial hybrid cell (cybrid) technology [45]. In rats, a reduction in the aerobic capacity is concomitant with a decrease in the amount of proteins required for mitochondrial biogenesis and oxidative function in skeletal muscle, and with an increase in cardiovascular risk factors [46].

The ethiopathogenic complexity of stroke is paralleled by that of mitochondrial disorders, probably in part due to their dual genetic control (mitochondrial and nuclear) and interplay with the environment. A small minority of complex I to IV subunits are mtDNA-encoded and produced, while the majority of subunits are nuclear-encoded and transported into the organelle. It is likely that mtDNA polymorphisms and haplogroups act synergistically with nuclear genetic factors and environmental components, and therefore mtDNA-encoded gene/nuclear-encoded gene and mtDNA-encoded gene/environment epistatic interactions may explain a larger fraction of the ischemic stroke heritability.

## Conclusion

We found suggestive evidence for association of the mitochondrial haplogroup H1 with ischemic stroke. For a deeper insight of the role of mtDNA variants in ischemic stroke, the full-sequencing of the molecule and the replication of the same polymorphisms in a large, well-matched, independent dataset are mandatory. If replicated in other populations, these influences on ischemic stroke risk are a relevant matter of public health given that haplogroups H1, pre-HV/HV, U, and U5 represent about 20% of the European population.

## Competing interests

The authors declare that they have no competing interests.

## Authors' contributions

MC, JMF, AMV, and SAO participated in study design. BVF, IA, GG, LG, MC, MVB, RMS, ANP, RT, CF, JRF, MRS, JPG, IM, GL, and JMF contributed to sample collection. AR, BVF, TK, HM, IA and GG generated genotyping data. AR, TK, HM, AMV and SAO assisted in statistical analysis. AR, TK, HM, JMF, AMV and SAO interpreted the results. All authors intervened in manuscript preparation and/or critical revision, read and approved the final manuscript.

## Pre-publication history

The pre-publication history for this paper can be accessed here:


